# Estimating Risk of Introduction of Ebola Virus Disease from the Democratic Republic of Congo to Tanzania: A Qualitative Assessment

**DOI:** 10.3390/epidemiologia3010007

**Published:** 2022-02-11

**Authors:** Sima Rugarabamu, Janeth George, Kennedy M. Mbanzulu, Gaspary O. Mwanyika, Gerald Misinzo, Leonard E. G. Mboera

**Affiliations:** 1 SACIDS Foundation for One Health, Sokoine University of Agriculture, Chuo Kikuu, Morogoro P.O. Box 3297, Tanzania; Janeth.george@sacids.org (J.G.); mbanzulu.kennedy@sacids.org (K.M.M.); gaspary.mwanyika@sacids.org (G.O.M.); gerald.misinzo@sacids.org (G.M.); lmboera@gmail.com (L.E.G.M.); 2 Department of Microbiology and Immunology, Muhimbili University of Health and Allied Sciences, Dar es Salaam P.O. Box 65001, Tanzania; 3 Department of Tropical Medicine, Infectious and Parasitic Diseases, University of Kinshasa, Kinshasa P.O. Box 747, Democratic Republic of the Congo; 4 Department of Medical Sciences and Technology, Mbeya University of Science and Technology, Mbeya P.O. Box 131, Tanzania

**Keywords:** risk assessment, Ebola virus disease, population movement, introduction, Tanzania

## Abstract

Between April 2018 and November 2020, the Democratic Republic of Congo (DRC) experienced its 11th Ebola virus disease (EVD) outbreak. Tanzania’s cross-border interactions with DRC through regular visitors, traders, and refugees are of concern, given the potential for further spread to neighboring countries. This study aimed to estimate the risk of introducing EVD to Tanzania from DRC. National data for flights, boats, and car transport schedules from DRC to Tanzania covering the period of May 2018 to June 2019 were analyzed to describe population movement via land, port, and air travel and coupled with available surveillance data to model the risk of EVD entry. The land border crossing was considered the most frequently used means of travel and the most likely pathway of introducing EVD from DRC to Tanzania. High probabilities of introducing EVD from DRC to Tanzania through the assessed pathways were associated with the viability of the pathogen and low detection capacity at the ports of entry. This study provides important information regarding the elements contributing to the risk associated with the introduction of EBV in Tanzania. It also indicates that infected humans arriving via land are the most likely pathway of EBV entry, and therefore, mitigation strategies including land border surveillance should be strengthened.

## 1. Introduction

Ebola virus disease (EVD) is a severe, often fatal zoonotic disease of humans, nonhuman primates, duikers, and bush pigs that have appeared sporadically since its initial recognition in the Democratic Republic of Congo (DRC) and South Sudan in 1976 [1,2]. Large numbers of EVD cases were later reported in 1994 and 1995 when the disease occurred in Uganda and the DRC, respectively [3,4]. Frequent large EVD outbreaks have since been reported from Gabon, the Republic of Congo, DRC, Uganda [5], Guinea, Liberia, Sierra Leone, and Nigeria [6,7]. There has been an increase in EVD outbreaks in Africa, probably because of increased contact of humans and wildlife due to extensive deforestation, hunting, mining, and human movements [8,9,10]. In the African Great Lakes Region, both DRC and Uganda have experienced several EVD outbreaks since 1976. Between 1979 and 2018, DRC and Uganda have experienced 10 and 5 EVD epidemics, respectively [11,12]. Although the incidence of EVD may be low compared to other infectious diseases in Africa, its high index of illness severity, high case fatality rate, and profound human suffering trigger public panic nationally and globally [13,14]. In addition, EVD outbreaks have resulted in pronounced socioeconomic and political impacts in the affected countries [15].

Travel, migration, displacement, and globalized trade have been described to contribute to the increased risk of infectious diseases significantly [16,17]. The West Africa EVD epidemic of 2014–2016 has shown clearly how interconnectedness and the cross-border nature of the threat have facilitated its transmission across bordering countries [18]. Trading has been an essential factor that links Tanzania and DRC [19]. In addition to trade that brings several Congolese to Tanzania, Tanzania has continued to grant asylum to Congolese refugees on a prima facie basis [19,20]. Currently, Tanzania hosts 82,000 refugees and asylum seekers from the DRC, most in the Kigoma Region in western Tanzania [20]. All these are likely to play a role in importing EVD from DRC to Tanzania.

Risk analysis has been used to assess the probability of introducing specific diseases and their consequences [21,22] to minimize disease occurrence. Epidemic risk analysis can predict potential serious outcomes and, therefore, the need for prompt responses [23]. There are two components of risk, namely likelihood and consequence. The likelihood is the probability of a disease entering, establishing, or spreading in the importing country, whereas the consequence is the effect on human or animal health, the environment, public welfare, and the economy [24,25]. The risk analysis process comprises four components: hazard identification, risk assessment, risk management, and risk communication [25]. Many current risk assessments are based on static elements that are inherent to the infectious disease. In an emerging infectious disease situation, climatic events and regional or local vulnerabilities are changeable, and the level of risk is often difficult to determine [25,26]. There is a need for risk assessment tools that incorporate changing risk elements during disease emergence [26].

On 1 June 2018, the World Health Organization (WHO) declared the 11th EVD outbreak a public health emergency of international concern given its potential for further outbound spread [27]. Despite several EVD outbreaks in the neighboring DRC and Uganda, Tanzania is yet to conduct EVD epidemic risk analysis and assess the likelihood of EVD importation. Risk analysis is critical in prioritizing a response to an epidemic as it provides crucial information on the potential impact of the disease outbreak [26]. The objective of this study was to estimate the probability of introducing EVD from DRC to Tanzania. The findings are expected to provide initial information for the most likely EVD entry pathways and, therefore, develop appropriate mitigation strategies.

## 2. Methods

### 2.1. Study Area and Context

Tanzania is one of the countries in the Great Lakes Region of Africa located between latitude 6°22′22.17″ S and longitude 34°53′32.94″ E. Neighboring countries include Burundi, the Democratic Republic of the Congo, Kenya, Malawi, Mozambique, Rwanda, Uganda, and Zambia. It has 59,734,218 people with an estimated growth rate of 3.0% [28]. One of its regions, namely Kigoma, located in the west, shares its land border with Burundi and DRC. Dar es Salaam has an international airport and seaport, through which most of the passengers from DRC import and export goods. The regional health system operates in a decentralized organization with public and private health service delivery [29]. Tanzania has about 42 ports of entry responsible for overseeing the implementation of International Health Regulations [30].

### 2.2. Data Collection and Tools

Data used to estimate the model input parameters were collected between April 2018 and November 2020 during the time of intense Ebola virus transmission and wide spread when the Democratic Republic of Congo (DRC) experienced its 11th Ebola virus disease (EVD) outbreak. Scientific literature, expert opinion, personal experience, and other sources of information were used to establish an understanding of agent (pathogen), host, and environmental factors that are important in epidemiologic aspects of EVD.

Patterns of arrivals from the DRC to Tanzania were studied by obtaining travel datasets from Tanzania Immigration Services, Ministry of Health, Tanzania Ports Authority, and Tanzania Civil Aviation Authority representing the most up-to-date available data to develop a list of potential pathways for release of the EVD into Tanzania from DRC. Information on the volume of travelers, travel time, direct and indirect connections, and reasons for entry was collected.

We additionally extracted information of arrivals from March 2016 to March 2017 to document the arrival patterns, volume, and destinations before the outbreak and observe any changes that would have been attributed to an outbreak. We also mapped the final destinations using ESRI ArcGIS version 10, indicating which cities receive direct and nondirect travelers from DRC, assuming all travelers from DRC have possible exposure to the virus. Other information such as traveler screening to capture those exposed and the time required to capture symptomatic travelers was obtained from published literature, online publications through internet searches (key terms: EVD risk*, EVD surveillance*, EVD prevalence*, EVD control*, EVD policy*, Ebola Virus Disease*) and specific databases such as PubMed and Google Scholar.

Finally, available data from governmental and public domain sources and other information (e.g., expert opinion) were obtained to evaluate the likelihood of each pathway introducing EVD to Tanzania and identify the regions at risk for possible exposure to EVD.

### 2.3. Qualitative Assessment

The methods used to conduct this risk assessment were based on the work of Kasari [31]. Evaluation of the following factors, both in the country of origin (DRC) and destination (Tanzania), was also conducted: disease status; population movements; and the legal framework, surveillance, and countries’ levels of connectivity to Ebola virus-affected areas but with constrained healthcare resources

#### 2.3.1. Risk Assessment Framework

The technical step in the assessment of the paths along which EVD can be introduced from DRC to Tanzania and establish an outbreak of disease in susceptible humans was done as follows: (a) reviewing agent, host, and environmental factors important in EVD, (b) modeling the pathway (outline the conceptual model and develop a list pathway), (c) evaluating the feasibility of each pathway, and (d) identifying the populations at risk for possible exposure to the EVD.

#### 2.3.2. Review of Agent, Host, and Environmental Factors Important in EVD

EVD was first reported in 1976 [2]. It is caused by an RNA virus of the *Filoviridae* family [8]. EVD is perhaps the most severe and feared of all viral hemorrhagic fevers. The virus replicates quickly and achieves an extremely high concentration in the cytoplasm of host cells, particularly those of the liver and other reticuloendothelial organs [32,33,34]. The infected individual typically has widespread severe cytopathologic changes in these tissues during the 2- to 7-day incubation period of this disease [35]. The virus survives well at ambient temperature and when frozen or lyophilized, but it is quite sensitive to acidic conditions and readily inactivated by lipid solvents (e.g., ether), detergents, and common disinfectants [36]. All of the known human pathogenic ebolaviruses are endemic only in sub-Saharan Africa. Recent evidence strongly implicates fruit bats as the ebolavirus reservoir, with human infection likely from inadvertent exposure to infected bat excreta or saliva [37]. Miners, spelunkers, forestry workers, and others are exposed to environments where bats typically roost are at risk [1]. Nonhuman primates, especially gorillas and chimpanzees, and other wild animals may become infected and serve as intermediate hosts that transmit *ebolavirus* to humans through contact with their blood and bodily fluids, usually associated with hunting and butchering [1,36,37]. These wild animals are presumably also infected by bats and develop a severe and usually fatal disease similar to human VHF [37]. *Zaire ebolavirus* has caused large die-offs of central chimpanzees and western lowland gorillas in central Africa [1]. Ebola virus outbreaks tend to occur at the end of the rainy season [38]. *Ebolavirus* remains the most transmissible of all VHFs. In most outbreaks, it appears that there is a single introduction or very few introductions from a zoonotic source into humans followed by nosocomial amplification in a setting of inadequate universal precautions, usually in rural areas of countries where civil unrest has decimated the healthcare infrastructure [38]. Ebola virus transmission occurs when the infectious agent has been amplified to reach the minimum logarithm of the virus (threshold). Humans are good at amplifying EBV [39]. Severe disease is generally associated with high viremia approximately 8 days after the onset of the disease, facilitating transmission into other humans [38,40]. EBV is not typically excreted into the atmosphere as an aerosol by an ill person [41]. Human-to-human transmission occurs through direct contact with contaminated blood or body fluids [39,40,41,42]. Infection probably occurs most often through oral or mucous membrane exposure in the context of providing care to sick family members in the community or patients in a healthcare institution or during funeral rituals that entail the touching of the corpse prior to burial [42].

Since 1976, when EVD was first detected in DRC, 34 outbreaks with 1–10,000 cases per outbreak have been reported in 11 countries in Africa [12]. The affected countries include the DRC, South Sudan, Uganda, Mali, Nigeria, Sierra Leone, Guinea, Liberia, South Africa, Gabon, and Côte d’Ivoire. More outbreaks have been reported in DRC in which physical factors such as geographic location and climatic conditions and more obvious social, economic, or political factors contribute to their occurrence.

#### 2.3.3. Scenario Pathways

**Figure 1:**
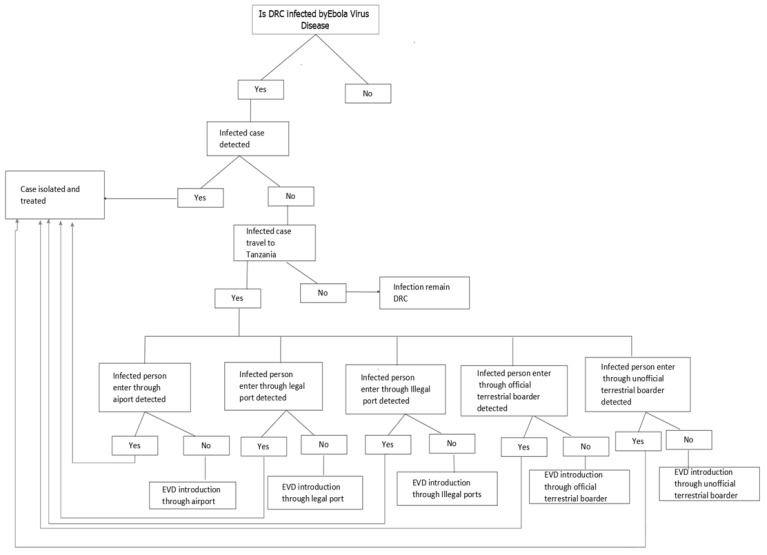
Scenario event pathways for the release of EVD to Tanzania.

A conceptual model representing a chain of potential events that may result in EVD entry was constructed (Figure 1). Individual exposure assessments were undertaken to define the probability of exposure to EVD through each of the five routes of entry outlined in the conceptual model. For each probability of exposure, the literature was reviewed to collate data that would enable a qualitative estimate of the probability to be derived. All influential factors for exposure to EVD for each route and the qualitative probability of exposure were assessed.

Following the exposure assessment, a consequence assessment was undertaken. The consequence was defined as the likelihood of the EVD spread, given the exposure. Relevant literature was sought that addressed the dependence of the consequence on healthcare capacity, employing national indicators of healthcare system capacity such as healthcare expenditures per head, physicians, and hospital beds per 1000 population. Countries’ levels of connectivity to Ebola virus-affected areas but with constrained healthcare resources were identified. Exposure and consequence were assessed for all routes. Predefined qualitative categories of all routes were classified as negligible (event is sufficiently low to be ignored), low (the event may occur in some cases), moderate (the event may occur in all cases), or high (event is likely to occur).

## 3. Results

### 3.1. Exposure Assessment

**Figure 2:**
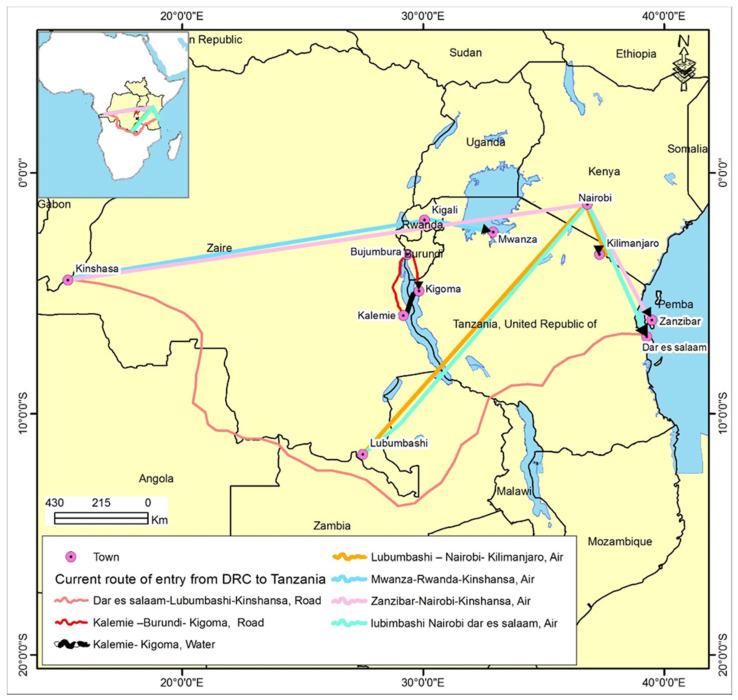
Current routes of entry from DRC to Tanzania.

The recent EVD outbreak has affected most of the North Kivu and Ituri provinces of DRC, less than 2000 km from the Tanzanian border. The physical pathways that travelers used were ground travel, water vessels, and air travel. The land border crossing was considered the most frequently used means of travel (highest), while water and air travel were deemed less frequently used (high). While Kigoma has direct connections with DRC regions, Mwanza, Dar es Salaam, Kilimanjaro, and Zanzibar airports were identified as indirect connections (Figure 2). Outbreak report analyses have determined that the outbreaks continue to occur in closer regions than seen in the past and on average, it takes 1–3 days to travel from the outbreak regions to the Tanzania–DRC border (Table 1). More than 15,000 arrivals from DRC to Tanzania occurred in 2018, translating to a net migration rate of 0.5 migrants per 1000 population [42]. This volume accounted for about two times the volume of arrival in 2017. The outbreak trend started with 1000 cases of EVD during the first eight months, which doubled with other new cases to an average notification of 75–100 cases per week. This was followed by a decline and remained comparatively low throughout the end of 2019 and early 2020.

Kigoma has about 20 ports of entry (PoEs). Of these, 8 are official and 12 are unofficial. The PoEs included 1 airport, 10 land border PoEs, and 9 water border PoEs on Lake Tanganyika and the Malagarasi River. The airport pathways use a computer-based border management information system (BMIS) to record human movements. Ten PoEs use official ledger books or notebooks to record entries and exits, while five PoEs do not record human movements.

Most people prefer to use unofficial PoEs since they do not have official government travel documents. Most frequently, unofficial PoEs were used during “event days”, such as market days. Immigration and port reports show that most people use the PoEs to cross in and out of the country for business-related reasons or social relations issues across the border. Some cross the border to seek and access healthcare services from facilities in Tanzania. All the official PoEs conduct screening services, while the informal PoEs rarely conduct screening. Thermal screenings of arrivals were reported to be conducted at the PoEs with available screening equipment and the required staff to perform the necessary screening. The official PoEs had a good capacity for screeners, personal protective equipment, screening infrastructure, and standard operating procedures.

Following the reports of the outbreak in DRC, Tanzania activated and operationalized coordination mechanisms for emergency management against EVD. This included sensitizing the community through leaders (village, influential people, religious) to report any EVD suspect case. Public education through mass media emphasized community standard case definition and the importance of early reporting to health facilities. The regional and district authorities were responsible for ensuring awareness campaigns were conducted for all front-line health workers on detecting and reporting of cases. However, laboratory capacity was a challenge as most facilities had inadequate capacities to detect viral infections within the required timeframe of less than 48 h.

### 3.2. Risk Assessment

During the study period, no reported restrictions were imposed on travel from DRC to Tanzania. Travelers using land and lake port pathways accounted for 2 times more volume than air travelers. Studies conducted to estimate the probability of infected persons departing the outbreak region projected that three travelers infected with the Ebola virus are likely to depart in one year if restrictions are instituted [43,44]. The projection used the number of active cases, which were defined as confirmed, probable, or suspected cases within the 21 days; country population estimates; and the monthly number of travelers pre-outbreak and calculated the expected numbers of Ebola virus exportations by multiplying the number of active case per country and months of traveling [45]. The method assumed a homogeneous distribution and constant prevalence of infection in the general population with equal risk of infection between travelers and nontravelers. To avoid uncertainties, sensitivity analysis was conducted to explore scenarios of increasing case burden [45]. Since during our study, there were no travel restrictions between DRC and Tanzania, we assumed an increasing number of infected person departures from outbreak regions; thus, if there is no effective health screening of travelers, there is a high possibility of EVD entry through all pathways.

Exit screening of travelers at points of exit would allow assessment of all travelers departing DRC. Usually, the large volume of travelers would have to be screened to identify travelers from DRC. Our study found travel times of 6–8 h flight and 1 to 2 days by road and water from DRC to Tanzania. Due to the incubation time of the Ebola virus (2–21 days), it is unlikely that an infected individual who was asymptomatic at exit screening would develop symptoms during their journey. The usefulness of entry screening in addition to exit screening might seem low. However, three factors urge the implantation of entry screening of passengers from DRC to Tanzania, especially the arrivals via land and water entries: (i) DRC is a resource-poor country in the midst of an emergency, and hence maximum efficacy screening might be limited without support from the international community [46]; (ii) the presence of unofficial border ports of entry means the absence of exit and entry screening may occur, hence posing a greater risk of Ebola disease entry; and (iii) direct contact with infected body fluids such as blood, sweat, saliva, sperm, vaginal fluids, urine, and sputum, or direct inoculation with contaminated tools such as needles, pins, and razor blades, results in transmission from one person to another. Nosocomial transmission has been documented using infected needles and syringes [11,40]. Person-to-person transmission is frequently at the root of large EVD epidemics, with caregivers in particular at risk [41]. During most epidemics, it has been reported that patients who became infected had been in contact with body fluids, direct physical contact with an infected person, and handling the body of an infected deceased person [42]. In addition, the disease was linked to the indirect transmission through sleeping on the same mat, participating in traditional hand washing during the funeral rite, and having a communal meal during the funeral service [43]. The above social and cultural behaviors have been documented among communities living at the DRC–Tanzania borders, thus making land and port travel at a higher risk of introducing EVD from DRC to Tanzania.

### 3.3. Entry of EVD

All national airports are potential entry pathways for humans infected with Ebola. Between 2018 and 2019, about 500,000 airline passengers from different African countries entered the country. Major arrivals were from Uganda, Zambia, Kenya, Rwanda, and countries with direct connection to DRC. The number of airline flights originating from non-EVD-endemic countries but picking up passengers from DRC could not be determined. On entering Tanzania, passengers from DRC completed a questionnaire as a requirement of inspection, with the majority indicating that they were visiting family members and friends; the questionnaire did not specifically ask where the family members or friends of these passengers lived. Most Congolese reside in Dar es Salaam, Morogoro, Kigoma, and Katavi. These states are probably at greater risk for the introduction of EVD in the event that one of these passengers who traveled specifically to visit family members or friends is viremic at the time of landing. In addition, Tanzania citizens traveled to DRC and the neighboring countries, respectively. During the time frame evaluated in this pathway analysis, no returning citizens or airline passengers originating from DRC or at-risk African countries were quarantined on arrival because of clinical signs of disease compatible with EVD.

#### 3.3.1. Air Pathway Feasibility

Air travel is a risk pathway for entry of people into Tanzania who may be viremic with EBV that was contracted while in DRC. Tanzania citizen passengers who are returning from visiting DRC and high-risk countries may also potentially contribute to the release of the virus in Tanzania. It is likely that passengers arriving from DRC would contribute to the release of the virus. Both DRC and Tanzania lack comprehensive surveillance programs.

Boats and cars departed to and from DRC almost daily during the time period evaluated in this risk analysis. Travelers using land and lake port pathways accounted for 2 times more volume than air travelers. The land border crossing was the most frequently used means of travel. Exit screening of travelers at points of exit would allow assessment of all travelers departing DRC. Usually, the large volume of travelers would have to be screened to identify travelers from DRC. From the interviews conducted at PoEs, immigration and port health officials stated that most people who come to Tanzania use the PoEs for business-related reasons, and others come to access health services or visit family and friends.

#### 3.3.2. Pathway Feasibility

With the large number of arrivals via land and port, it would be virtually impossible to screen each one. The virus could also be brought into Tanzania through entry points other than official PoEs. Experts agree that illegal entry routes should be acknowledged as the pathway of the highest potential. They were uncertain about the degree of importance placed on this pathway because of changing pattern presentation of the pathogens, the inability to predict illicit activities, and a lack of a system that can quickly detect and contain EVD. However, conventional wisdom dictates that efforts should not focus as much on preventing but rather on rapid detection and identification of disease incidents and establishing mechanisms for a quick response to an outbreak of EVD in Tanzania. Based on activities associated with the likely pathways, the population in Kigoma appears to be most vulnerable to exposure to EBV. The above finding shows that using a predefined scale, the probability of exposure to the Ebola virus from DRC for all routes is high.

## 4. Discussion

To our knowledge, this is the first risk assessment performed to evaluate the probability of introduction of EVD in Tanzania. Recent incursions of other infectious diseases have proven the fragility of existing public health surveillance and monitoring systems that could forecast and prevent the emergence of infectious diseases [45]. The introduction of dengue, severe acute respiratory syndrome, COVID-19, and others are examples that illustrate the need for an assessment of emerging diseases that can potentially pose the same risk of introduction and spread [46,47].

Risk assessment of an impending epidemic is likely to enhance decision-making and improve the planning of effective responses to minimize the spread of infectious diseases. A risk assessment has been considered a sound and valid method of estimating risk, having the advantage of being understood by many agents within different areas of expertise and disseminating knowledge [48]. Other studies have similarly evaluated the risk of the introduction of infectious disease agents in neighboring countries [49,50]. Although some authors consider that qualitative risk assessments can potentially overestimate risk by being limited in their approach to evaluating the occurrence of unwanted events accurately, our study has recognized their value and importance as one of the few appropriate methods sometimes available.

Unlike many current risk assessment tools based on static elements inherent to the infectious disease, this study’s methodology has considered dynamism. In an emerging infectious disease situation, climatic events, and regional or local vulnerabilities, we believe it is a comprehensive model that helped us reach conclusions and meet our objectives of facilitating relevant information. The first stage of the framework (review of agent, host, and environmental factors important in EVD) summarized the pathogen’s main characteristics under study and the aspects involved in the transmission cycle and pathogen viability. Due to its descriptive nature, this step required a more in-depth literature search and thorough reading of the current body of knowledge, which allowed us to confidently answer the questions that would move us forward in the framework.

This study identified that both DRC and Tanzania use thermal scans for screening. The effectiveness of this method can be challenged by the fact that peak viremia or assumed infectivity occurs within eight days of disease onset, a time that coincides with the severity of symptoms [51]. A person with an asymptomatic infection and in incubation period does not appear to be infectious. Thus, case patient isolation within 2–7 days of infection can limit transmission [52,53,54]. The risk of transmission during the incubation period or from asymptomatic persons is negligible. However, a high risk of silent spread by asymptomatic persons has been speculated. A case of Ebola was reported due to blood transfusion from a donor who was asymptomatic [55], and infected persons who remained asymptomatic played a significant role in the recent epidemics [56,57,58]. Because of the high risk of silent spread by asymptomatic persons, it is imperative that testing programs are supplemented with conventional diagnostic testing for both symptomatic and asymptomatic persons from outbreak regions.

This study suggests that both active screening and movement monitoring of arrivals should go hand in hand to limit exposure and spread from DRC to Tanzania. Case detection could be a useful strategy for delaying the entry of EVD into Tanzania and limiting the opportunity for the virus to spread. However, considering the available screening methods currently used and the long incubation period of EVD, there is the possibility of an imported case being brought to Tanzania by an asymptomatic person. To effectively intensify screening without causing much of a burden on the health system, the provision of information to arriving travelers and effective communication with local communities and health personnel need to be emphasized.

Regarding the entry pathways, we considered three main entryways by which EVD could be introduced to Tanzania. Air travelers from DRC have no direct flight to Tanzania. Thus, the high probabilities of introducing EVD through this pathway are associated with the viability of the pathogen and low detection capacity at the ports of entry such as Kigoma, Mwanza, and Dar es Salaam that have an active connection and cross-border movement with DRC. The possibility of infected humans entering Tanzania may be of public health concern. Although a large number of probabilities have been considered, these are not exhaustive. For example, with limited data, we could not examine all possible risk factors. Although imports seem to occur from few regions, there is a high degree of uncertainty associated with the entry points because there are several possible pathways, including entry via neighboring countries other than DRC. The release assessment considered at-risk groups by assuming exposure to EVD is evenly distributed to the community. This may impact the overall probability of release. The travel patterns, vulnerabilities of the country, epidemic dynamic, and risk communication greatly simplified this risk assessment while still identifying those pathways of a higher likelihood of infection. However, it is recognized that the under-recording of arrivals will impact the classification of entry points by the active connections [59]. Therefore, it is essential to review the situation periodically and, if necessary, adjust the region groupings accordingly. Where possible, heterogeneity of entry points should be considered, especially with their past and current situation and available health services.

Tanzania and DRC have connectivity with economic benefits that may suffer consequences once travel restrictions are imposed based on the findings of this study. Targeted programs to inform and educate populations on how to detect symptoms and avoid infection should be implemented. An additional strategy would be advisable for individuals before traveling, emphasizing a self-imposed 21-day monitoring period post-arrival for high-risk groups such as those from funeral attendance.

Countries have been screening travelers arriving at their borders for centuries for their security, health, and economic interest. However, in a world where the benefits of interconnectedness and the risks of interdependence are inextricably linked, member nations’ public health responses to global threats must be commensurate with and limited to public health risks and prevent unnecessary interruption to international passenger traffic and trade, as per the best available evidence.

## 5. Conclusions

Despite the limitations of data and comprehensive information, this work provides essential information on the risk of EVD introduction in Tanzania. In conclusion, this study estimated the risk of introducing EVD from DRC to Tanzania as high. Infected humans arriving via land are the most likely source of EVD introduction, and therefore, mitigation strategies should be directed towards this pathway. Frequent EVD outbreaks in closer regions urge the intensification of the surveillance system, including screening and registering travelers within all the border entry points to prevent disease importation. Equally important, there is a need for strengthening the sensitization and awareness campaigns focusing on cross-border communities. The border healthcare facilities need to be well equipped with all measures to ensure a high disease containment capacity.

## Figures and Tables

**Table 1:** Year of the outbreak, place of occurrence, and estimated time and distance to Tanzania.

Year	Place of Occurrence	Travel Time	Distance to Tanzania	Means of Transport
1976	Yambuku	2 days	2497	Road
1977	Tandala	3 days	3743	Road
1995	Kikwit	2 days	3778	Road/Air
2007	Mweka	2 days	3581	Road/Air
2008	Luebo	3 days	3452	Road/Air
2012	Isiro	1–2 days	2398	Road/Air
2014	Boende	2–3 days	3714	Road/Air
2017	Likati	2–3 days	3032	Road/Air
2018	Beni	1 day	1875	Road/Air
2019	Beni/Isiro	1–3 days	1875–2398	Road/Air/Ferry

## Data Availability

The data that support the finding of this study are available on request from the corresponding author.
